# Single-event multilevel surgery in cerebral palsy: Value added by a co-surgeon

**DOI:** 10.1097/MD.0000000000026294

**Published:** 2021-06-18

**Authors:** Nickolas J. Nahm, Meryl Ludwig, Rachel Thompson, Kenneth J. Rogers, Ahmet Imerci, Kirk W. Dabney, Freeman Miller, Julieanne P. Sees

**Affiliations:** a Department of Orthopaedic Surgery, University of Nebraska, Omaha, NE; b Pediatric Specialists of Virginia, Fairfax, VA; c Department of Orthopaedic Surgery, David Geffen School of Medicine at UCLA,Orthopaedic Institute for Children, Los Angeles, CA; d Department of Orthopaedic Surgery, Nemours/Alfred I. duPont Hospital for Children, Wilmington, DE, USA; e Department of Orthopaedics and Traumatology, Mugla Sitki Kocman University, Merkez, Mugla, Turkey; f National Academy of Medicine Fellowship, American Osteopathic Association, Chicago, IL, USA.

**Keywords:** cerebral palsy, co-surgeon, lower extremity osteotomy, single-event multilevel surgery

## Abstract

The aim of this study was to compare outcomes for single-event multilevel surgery (SEMLS) in cerebral palsy (CP) performed by 1 or 2 attending surgeons.

A retrospective review of patients with CP undergoing SEMLS was performed. Patients undergoing SEMLS performed by a single senior surgeon were compared with patients undergoing SEMLS by the same senior surgeon and a consistent second attending surgeon. Due to heterogeneity of the type and quantity of SEMLS procedures included in this study, a scoring system was utilized to stratify patients to low and high surgical burden. The SEMLS events scoring less than 18 points were categorized as low burden surgery and SEMLS scoring 18 or more points were categorized as high burden surgery. Operative time, estimated blood loss, hospital length of stay, and operating room (OR) utilization costs were compared.

In low burden SEMLS, 10 patients had SEMLS performed by a single surgeon and 8 patients had SEMLS performed by 2 surgeons. In high burden SEMLS, 10 patients had SEMLS performed by a single surgeon and 12 patients had SEMLS performed by 2 surgeons. For high burden SEMLS, operative time was decreased by a mean of 69 minutes in cases performed by 2 co-surgeons (*P* = 0.03). Decreased operative time was associated with an estimated savings of $2484 per SEMLS case. In low burden SEMLS, a trend toward decreased operative time was associated for cases performed by 2 co-surgeons (182 vs 221 minutes, *P* = 0.11). Decreased operative time was associated with an estimated savings of $1404 per low burden SEMLS case. No difference was found for estimated blood loss or hospital length of stay between groups in high and low burden SEMLS.

Employing 2 attending surgeons in SEMLS decreased operative time and OR utilization cost, particularly in patients with a high surgical burden. These findings support the practice of utilizing 2 attending surgeons for SEMLS in patients with CP.

Level of Evidence: Level III

## 1 Introduction

Cerebral palsy (CP) is caused by an injury to the developing brain and frequently results in upper extremity contractures and lower extremity deformities including hip dysplasia, long bone torsion, knee flexion contractures, ankle equinus contractures, and foot deformities.^[1,2]^ Corrective orthopedic surgery is required for many affected children with the overall goals of maintaining comfort and preserving function.^[3]^ Introduced in 1985 by Norlin and Tkaczuk,^[4]^ single-event multilevel surgery (SEMLS) is the preferred strategy for extremity surgery in children with CP.^[5–8]^ In a randomized control trial, the Melbourne group demonstrated improved gait parameters in children undergoing SEMLS compared with a control group treated with resistance training.^[5]^ Improved gait parameters in the SEMLS group were maintained at follow-up of 5 years.^[6]^ The goal of SEMLS is to avoid multiple hospitalizations, convalescence periods, and cycles of rehabilitation.^[9,10]^

By definition, SEMLS includes multiple procedures and is often performed by 2 attending co-surgeons.^[11]^ The concept of dual attending surgeons is previously studied in pediatric spine surgery.^[12–17]^ These studies demonstrate shorter operative time, decreased blood loss, and shorter hospital length of stay. However, there is limited data supporting the use of 2 attending surgeons in SEMLS for CP surgery.

The challenge in studying SEMLS performed for lower extremity deformity in patients with CP is both the heterogeneity of procedures and the variance in the number of procedures included among patients.^[7]^ Previous studies controlled for this either by examining 1 specific procedure performed in the context of SEMLS^[18–20]^ or by quantifying the amount of surgery based on postoperative rehabilitation demands of each type of procedure.^[21]^ However, these approaches do not reflect the operative time or resources required to complete SEMLS. As such, we aimed to develop a new system for quantifying the amount of surgery based on operative time and operating room (OR) resources required by our cohort to allow for a more equitable comparison between surgical cases.

To determine the impact of 2 attending surgeons compared with 1 attending surgeon performing SEMLS, we aimed to compare outcomes between patients undergoing SEMLS, dividing patients into low and high surgical burden groups, utilizing this new system. We hypothesized that surgery performed by 2 attending surgeons required less operative time, resulting in decreased blood loss, shorter hospital length of stay, and decreased costs in both cohorts.

## 2 Materials and methods

Retrospective review was approved by the Institutional Review Board of Nemours Children's Health System, and charts were reviewed for SEMLS performed between 2010 and 2017. Patients were classified into either the single surgeon or 2 surgeon group. The single surgeon group consisted of patients who underwent SEMLS performed by 1 of the senior authors. The 2 surgeon group consisted of patients who underwent SEMLS with a surgical team consisting of the same senior author and another senior author operating as co-surgeons. Inclusion criteria for each group were a diagnosis of CP, age 21 years old or younger at the time of surgery, and at least 1 foot procedure included in the SEMLS event. Subjects in each group were matched on type of concurrent procedures performed, functional level (within 1 level of the Gross Motor Function Classification System, [GMFCS]),^[22]^ and age (within 1 year at the time of surgery). Exclusion criteria were SEMLS events not including a foot procedure and subjects unable to be matched as defined. Primary outcomes of interest were operative time, estimated blood loss, and hospital length of stay. Operative time was from skin incision to wound closure and excluded time required for anesthesia induction and wake-up. Other patient follow-up data were not collected.

Due to the heterogeneity of procedures and variation in the amount of surgery inherent to SEMLS, patients in the single surgeon and 2 surgeon groups were further stratified by the amount of surgery performed to either a low surgical burden group or a high surgical burden group. To stratify patients by the amount of surgery performed, commonly performed SEMLS procedures were assigned a point value (Table 1). Point values had a range from 1 to 4 and were assigned based on the amount of effort (time, technical difficulty, and amount of assistance required) to complete the procedure, as perceived by 3 pediatric orthopedic surgeons. Point assignment for each procedure was determined based on consensus among 3 practicing pediatric orthopedic surgeons.

**Table 1:** Score allocation to assign surgical burden for procedures performed during single-event multilevel surgery.

Procedure	Score
Proximal femoral osteotomy	4
Distal femoral extension osteotomy	4
Pelvic osteotomy	3
Rectus transfer	2
Posterior knee capsulotomy	2
Tibial derotation osteotomy	2
Rectus resection	1
Foot osteotomy or fusion	2
Soft tissue lengthening, advancement, recession, or plication	1

Points were assigned to all procedures performed for each patient, and the points were tallied to determine a surgical burden score. Patients with less than 18 points were assigned to the low burden group, and patients with 18 or more points were assigned to the high burden group. This threshold was selected to most evenly divide the cohort into low and high burden groups. Two pediatric orthopedic surgeons assigned points for each patient, and inter-rater reliability was calculated. Discrepancies were resolved by 1 of the 2 senior surgeons for the purposes of assigning patients to cohorts for this study. One rater repeated classification 2 months after initial scoring to calculate intra-rater reliability.

After subdividing patients into high and low burden groups, single and dual surgeon SEMLS were compared by operative time, estimated blood loss, and hospital length of stay. Cost of OR time was estimated at $37.45 per minute based on an analysis of 302 hospitals in California in 2014.^[23]^ Categorical variables were compared with Fisher exact test or Chi-square testing as appropriate, and continuous variables were compared with Student *t* test for parametric data and Mann–Whitney test for non-parametric data. Intraclass coefficients were calculated to determine intra-rater and inter-rater reliability. Intraclass coefficients between 0.75 and 1.00 were interpreted as excellent, 0.60 and 0.74 as good, 0.40 and 0.59 as fair, and less than 0.40 as poor.^[24]^*P* values <0.05 were considered statistically significant.

## 3 Results

A total of 40 patients were included for analysis: 20 patients in the single surgeon cohort and 20 in the 2 surgeon cohort. Mean age at the time of surgery was 11.9 years (range: 6–16 years) for the single surgeon cohort and 13.7 years (range: 6–19 years) for the 2 surgeon cohort (*P* > 0.05). The GMFCS level distribution in the single surgeon cohort was 1 (GMFCS I), 3 (GMFCS II), 9 (GMFCS III), 4 (GMFCS IV), and 3 (GMFCS V). For the 2 surgeon cohort, GMFCS distribution was 1, 3, 8, 4, and 4 patients, respectively. The most frequently performed surgery was osseous foot surgery (including osteotomies and fusions; n = 149 in the entire cohort) followed by soft-tissue lengthening (n = 146 in the entire cohort) (Table 2). On average, each patient had approximately 10 procedures included in his or her SEMLS.

**Table 2:** Procedures performed in the low burden and high burden groups.

		Low burden		High burden	
	Entire cohort (n = 40)	Single surgeon (n = 10)	Two surgeons (n = 8)	Single surgeon (n = 10)^∗^	Two surgeons (n = 12)^†^
Proximal femoral osteotomy	29	5	6	10	8
Distal femoral extension osteotomy	10	2	2	2	4
Pelvic osteotomy	16	2	2	6	6
Rectus transfer	13	3	0	10	0
Posterior knee capsulotomy	4	1	0	0	3
Tibial derotation osteotomy	12	4	1	4	3
Rectus resection	11	3	2	2	4
Foot osteotomy or fusion	149	22	21	45	61
Soft tissue lengthening, advancement, recession, or plication	146	31	18	48	49
Total	395	73	52	129	141

The scoring system for classifying the burden of surgery had excellent inter- and intra-rater agreement with intraclass correlation coefficients of 0.925 and 0.975, respectively. In assigning patients to low or high surgical burden groups, the scoring system also had excellent inter-rater and intra-rater reliability with kappa scores of 0.975 for both. In the single surgeon group, 10 patients underwent low burden surgery with, on average, 7 procedures per SEMLS event and a median surgical burden score of 14 (range: 9–16). Ten patients in the single surgeon group underwent high burden surgery with, on average, 13 procedures per SEMLS event and a median surgical burden score of 20.5 (range: 18–32). In the 2 surgeon group, 8 patients underwent low burden surgery with, on average, 7 procedures per SEMLS event and a median surgical burden score of 15 (range: 10–17). Twelve patients in the 2 surgeon group underwent high burden surgery with, on average, 12 procedures per SEMLS event and a median surgical burden score of 21 (range: 18–31). Overall, the low and high surgical burden groups were well matched in the single surgeon and 2 surgeon cohorts.

Among patients who had low burden surgery, there was a trend toward decreased operative time favoring the 2 surgeon cohort by 39 minutes (182 vs 221 minutes, *P* = 0.11, Table 3), but this did not reach statistical significance. This decrease in operative time was associated with an estimated savings of $1460.55 per SEMLS case in OR utilization. No difference was detected in estimated blood loss or hospital length of stay. In the high burden surgery group, operative time was decreased by 69 minutes, on average, in the 2 surgeon group (247 minutes) compared with the single surgeon group (316 minutes, *P* = 0.03). This was associated with an estimated savings of $2584.05 per SEMLS case in OR utilization costs in the 2 surgeon group. No difference was detected in the high burden group with respect to estimated blood loss or hospital length of stay.

**Table 3:** Primary outcome variables in low burden and high burden surgical groups.

	Low burden			High burden		
	One surgeon	Two surgeons	*P*	One surgeon	Two Surgeons	*P*
Operative Time (min)	221	182	0.11	316	247	0.03
Estimated blood loss (mL)	357	252	0.69	264	499	0.22
Hospital length of stay (days)	4.5	3.8	0.22	5.5	5.1	0.42

## 4 Discussion

Among patients undergoing high burden SEMLS, employing 2 attending surgeons significantly decreased operative time and associated OR utilization cost, supporting the notion of co-surgery for this population. In the low burden group, a trend toward decreased operative time was detected in the 2 surgeon group, but this was likely underpowered to reach statistical significance.

To the best of our knowledge, this is the first report on the impact of co-surgery in this patient population for SEMLS. Previous studies examining the use of 2 attending surgeons in pediatric orthopedics focused on spine surgery. These authors similarly report the value of a 2 surgeon model for complex surgeries and patient populations.^[12–17]^ In a prospective analysis, Shrader et al. found decreased operative time, blood loss, and complications in patients with CP undergoing posterior spinal fusion performed by 2 attending level co-surgeons compared with single surgeon teams.^[12]^ In another prospective study of patients with adolescent idiopathic scoliosis undergoing posterior spinal fusion, Chan and Kwan found that surgery performed by 2 surgeons results in decreased operative time, blood loss, postoperative morphine usage, and hospital length of stay compared with single surgeon teams.^[14]^ These results were mirrored in subgroups of patients with Lenke 1 and 2 curves.^[15,16]^ Similarly, Abousamra et al. found decreased operative time and blood loss in spine surgery performed by 2 attending surgeons as compared with a single surgeon in patients with CP.^[13]^ Our study also demonstrated decreased operative time with 2 attending surgeons in SEMLS. However, no difference was detected for estimated blood loss likely due to widespread tourniquet use for the majority of extremity procedures included in this study.

A previous study from our institution examining StepWatch data in children with CP created a practical method for quantifying the amount of surgery performed during SEMLS.^[21]^ This system categorized patients from 1 (lowest surgical burden) to 5 (highest surgical burden) based on a number of factors including whether unilateral or bilateral surgery was performed, whether bony or soft-tissue procedures were performed, and the number of procedures performed. When applying this system to the patients in our cohort, all patients were rated at the highest surgical burden, which did not allow for patient stratification. Due to this ceiling effect, we created an alternative method for quantifying the amount of surgery that allowed for stratification into low and high surgical burden groups. This classification system had excellent inter- and intra-rater reliability, with the potential for broad applicability in SEMLS research. However, this scoring system requires extensive continued study, including application to a larger cohort of patients with CP undergoing SEMLS to determine external validity. Further, selecting 18 as a threshold for surgical burden quantification was useful in this cohort, but this threshold may not be applicable in other cohorts undergoing more or less surgery. Rather, this classification system may be most appropriately utilized as a continuous variable for stratification in larger, comparative studies. Nevertheless, the advantage of this system is that a score is assigned with no possibility of a ceiling effect.

Our study relied on the results of a robust analysis of over 300 hospitals in California to determine the cost of OR utilization.^[23]^ The authors of this study found an OR utilization cost of $36.14 per minute for the ambulatory setting and $37.45 per minute for the inpatient setting. Our institution is an inpatient pediatric facility, and we elected to utilize $37.45 per minute for our study. Determining the cost of medical care is notoriously difficult,^[25]^ and we acknowledge that further study is required. Local financial data is needed to obtain more accurate estimates of savings associated with shorter operative times. However, estimates in the literature for OR cost range from $7 to $100/minute.^[26–29]^ Therefore, utilizing a standard estimate from multiple institutions in the California study may give a more accurate representation of OR cost across centers nationally. Additionally, the use of a 2 surgeon team has been described by Ludwig et al,^[30]^ where the authors note that although a 2 surgeon team may lead to increased mean surgeon charges, the use of 2 surgeons demonstrated an overall decrease in OR and anesthesia charges. Although the potential opportunity loss is there for the second surgeon, there could also be opportunity gain for more cases to be performed in a single OR in a single day.

This study was limited by its retrospective nature and small sample size. A trend toward shorter operative time was found in the low surgical burden group, but the study was likely underpowered to detect a statistically significant difference. Furthermore, the results of this study may not be applicable to centers that are not familiar with a 2 attending surgeon model. A second attending surgeon is most valuable when a separate set of instruments and additional assistants are available. The difference in operative time may be less substantial when these resources are not available. In addition, the coding and billing department should be familiar with billing for surgery performed by co-surgeons to maximize the financial sustainability of this model.

Future study is required to examine the impact of a 2 surgeon model on surgeon fatigue. Each SEMLS case in this study had an average of approximately 10 procedures per patient with the longest case requiring nearly 7 hours. We hypothesize that performing this much surgery by a single surgeon may be associated with surgeon fatigue, which may affect the surgical outcome and lead to surgeon burnout. This type of study should be performed in a prospective manner with longer follow-up and should also include functional and health-related quality of life outcomes. Finally, robust cost analysis in a prospective study may offer more information about resource utilization.

Operative times appear to improve with the addition of a second attending surgeon, and this may potentially translate into improved patient-reported outcomes, but lost opportunity costs associated with the utilization of a second surgeon participating in the care of 1 patient need to be carefully evaluated in future studies. Within the limits of the available data, this current study supports the practice of utilizing 2 attending surgeons in high burden SEMLS for patients with CP. The benefit of a second attending surgeon in low burden surgery is less apparent but may be justified given the trend toward decreased operative time with 2 attending surgeons.

## Author contributions

**Conceptualization:** Nickolas Nahm, Meryl Ludwig, Rachel Thompson, Kirk Dabney, Freeman Miller, Julieanne P. Sees.

**Data curation:** Nickolas Nahm, Meryl Ludwig, Rachel Thompson, Ahmet Imerci, Julieanne P. Sees.

**Formal analysis:** Nickolas Nahm, Rachel Thompson, Kenneth Rogers, Ahmet Imerci, Freeman Miller, Julieanne P. Sees.

**Investigation:** Nickolas Nahm, Freeman Miller, Julieanne P. Sees.

**Methodology:** Nickolas Nahm, Freeman Miller, Julieanne P. Sees.

**Project administration:** Julieanne P. Sees.

**Resources:** Nickolas Nahm, Kenneth Rogers, Julieanne P. Sees.

**Validation:** Nickolas Nahm, Kenneth Rogers, Freeman Miller, Julieanne P. Sees.

**Writing – original draft:** Nickolas Nahm, Kenneth Rogers, Freeman Miller, Julieanne P. Sees.

**Writing – review & editing:** Nickolas Nahm, Rachel Thompson, Kenneth Rogers, Ahmet Imerci, Kirk Dabney, Freeman Miller, Julieanne P. Sees.

## References

[1] Rosenbaum P Paneth N Leviton A. A report: the definition and classification of cerebral palsy April 2006. Dev Med Child Neurol Suppl 2007;109:08–14. 17370477

[2] Graham HK Rosenbaum P Paneth N. Cerebral palsy. Nat Rev Dis Primers 2016;2:15082. 27188686 10.1038/nrdp.2015.82 PMC9619297

[3] Narayanan UG. Management of children with ambulatory cerebral palsy: an evidence-based review. J Pediatr Orthop 2012;32 Suppl 2:S172–81. 22890458 10.1097/BPO.0b013e31825eb2a6

[4] Norlin R Tkaczuk H. One-session surgery for correction of lower extremity deformities in children with cerebral palsy. J Pediatr Orthop 1985;5:208–11. 3988925

[5] Thomason P Baker R Dodd K. Single-event multilevel surgery in children with spastic diplegia: a pilot randomized controlled trial. J Bone Joint Surg Am 2011;93:451–60. 21368077 10.2106/JBJS.J.00410

[6] Thomason P Selber P Graham HK. Single event multilevel surgery in children with bilateral spastic cerebral palsy: a 5 year prospective cohort study. Gait Posture 2013;37:23–8. 22818117 10.1016/j.gaitpost.2012.05.022

[7] McGinley JL Dobson F Ganeshalingam R Shore BJ Rutz E Graham HK. Single-event multilevel surgery for children with cerebral palsy: a systematic review. Dev Med Child Neurol 2012;54:117–28. 22111994 10.1111/j.1469-8749.2011.04143.x

[8] Schranz C Kruse A Kraus T Steinwender G Svehli M. Does unilateral single-event multilevel surgery improve gait in children with spastic hemiplegia? A retrospective analysis of a long-term follow-up. Gait Posture 2017;52:135–9. 27907872 10.1016/j.gaitpost.2016.11.018

[9] Rodda JM Graham HK Nattrass GR Galea MP Baker R Wolfe R. Correction of severe crouch gait in patients with spastic diplegia with use of multilevel orthopaedic surgery. J Bone Joint Surg Am 2006;88:2653–64. 17142416 10.2106/JBJS.E.00993

[10] Stout JL Gage JR Schwartz MH Novacheck NF. Distal femoral extension osteotomy and patellar tendon advancement to treat persistent crouch gait in cerebral palsy. J Bone Joint Surg Am 2008;90:2470–84. 18978417 10.2106/JBJS.G.00327

[11] Georgiadis AG Schwartz MH Walt K Ward ME Kim PD Novacheck TF. Team approach: single-event multilevel surgery in ambulatory patients with cerebral palsy. JBJS Rev 2017;5:e10. 10.2106/JBJS.RVW.16.00101 28832346

[12] Shrader MW Wood W Falk M Segal LS Boan C White G. The effect of two attending surgeons on the outcomes of posterior spine fusion in children with cerebral palsy. Spine Deform 2018;6:730–5. 30348351 10.1016/j.jspd.2018.03.002

[13] Abousamra O Duque Orozco MDP Rogers KJ Miller F Sees JP. Effect of the first surgical assistant level on surgical outcome after spinal fusion in cerebral palsy. Del Med J 2019;91:114–6.

[14] Chan CYW Kwan MK. Perioperative outcome in posterior spinal fusion for adolescent idiopathic scoliosis: a prospective study comparing single versus two attending surgeons strategy. Spine (Phila PA 1976) 2016;41:E694–9. 26656053 10.1097/BRS.0000000000001349

[15] Kwan MK Chan CYW. Does a dual attending surgeon strategy confer additional benefit for posterior selective thoracic fusion in Lenke 1 and 2 adolescent idiopathic scoliosis (AIS)? A prospective propensity matching score analysis. Spine J 2017;17:224–9. 27609611 10.1016/j.spinee.2016.09.005

[16] Kwan MK Chiu CK Chan CYW. Single vs two attending senior surgeons: assessment of intra-operative blood loss at different surgical stages of posterior spinal fusion surgery in Lenke 1 and 2 adolescent idiopathic scoliosis. Eur Spine J 2017;26:155–61. 27734195 10.1007/s00586-016-4803-y

[17] Bauer JM Yanamadala V Shah SA Sethi RK. Two surgeon approach for complex spine surgery: rationale, outcome, expectations, and the case for payment reform. J Am Acad Orthop Surg 2019;27:e408–13. 30300215 10.5435/JAAOS-D-17-00717

[18] Firth GB Passmore E Sangeux M. Multilevel surgery for equinus gait in children with spastic diplegic cerebral palsy: medium-term follow-up with gait analysis. J Bone Joint Surg Am 2013;95:931–8. 23677361 10.2106/JBJS.K.01542

[19] Dreher T Buccoliero T Wolf SI. Long-term results after gastrocnemius-soleus intramuscular aponeurotic recession as a part of multilevel surgery in spastic diplegic cerebral palsy. J Bone Joint Surg Am 2012;94:627–37. 22488619 10.2106/JBJS.K.00096

[20] Truong WH Rozumalski A Novacheck TF Beattie C Schwartz MH. Evaluation of conventional selection criteria for psoas lengthening for individuals with cerebral palsy: a retrospective, case–controlled study. J Pediatr Orthop 2011;31:534–40. 21654462 10.1097/BPO.0b013e31821f8ee3

[21] Niiler TA Nicholson K Fischer L Lennon N. Factors influencing post-surgical variability in StepWatch data in youth with cerebral palsy. Gait Posture 2019;72:234–8. 31284160 10.1016/j.gaitpost.2019.06.017

[22] Palisano R Rosenbaum P Walter S Russell D Woo E Galuppi B. Development and reliability of a system to classify gross motor function in children with cerebral palsy. Dev Med Child Neurol 1997;39:214–23. 9183258 10.1111/j.1469-8749.1997.tb07414.x

[23] Childers CP Maggard-Gibbons M. Understanding costs of care in the operating room. JAMA Surg 2018;153:e176233. 29490366 10.1001/jamasurg.2017.6233 PMC5875376

[24] Cicchetti DV. Guidelines, criteria, and rules of thumb for evaluating normed and standardized assessment instruments in psychology. Psychol Assess 1994;6:284–90.

[25] Kaplan RS Porter ME. The big idea: how to solve the cost crisis in health care. Harvard Business Review [online]. September 2011. Available at: https://hbr.org/2011/09/how-to-solve-the-cost-crisis-in-health-care. Accessed April 10, 2020. 21939127

[26] Brown JK Campbell BT Drongowski RA. A prospective, randomized comparison of skin adhesive and subcuticular suture for closure of pediatric hernia incisions: cost and cosmetic considerations. J Pediatr Surg 2009;44:1418–22. 19573672 10.1016/j.jpedsurg.2009.02.051

[27] Ting NT Moric MM Della Valle CJ Levine BR. Use of knotless suture for closure of total hip and knee arthroplasties: a prospective, randomized clinical trial. J Arthroplasty 2012;27:1783–8. 23146366 10.1016/j.arth.2012.05.022

[28] Eggers MD Fang L Lionberger DR. A comparison of wound closure techniques for total knee arthroplasty. J Arthroplasty 2011;26:1251.e1-4–8.e1-48. 21531114 10.1016/j.arth.2011.02.029

[29] Lukish J Powell D Morrow S Cruess D Guzzetta P. Laparoscopic appendectomy in children: use of the endoloop vs the endostapler. Arch Surg 2007;142:58–61. discussion 62. 17224501 10.1001/archsurg.142.1.58

[30] Ludwig AT Inampudi L O’Donnell MA Kreder KJ Williams RD Konety BR. Two-surgeon versus single-surgeon radical cystectomy and urinary diversion: impact on patient outcomes and costs. Urology 2005;65:488–92. 15780361 10.1016/j.urology.2004.10.012

